# Machine Learning-Based Sensor Data Modeling Methods for Power Transformer PHM

**DOI:** 10.3390/s18124430

**Published:** 2018-12-14

**Authors:** Anyi Li, Xiaohui Yang, Huanyu Dong, Zihao Xie, Chunsheng Yang

**Affiliations:** 1 College of Information Engineering, Nanchang University, Nanchang 330031, China; 6101116067@email.ncu.edu.cn (A.L.); 6002115114@email.ncu.edu.cn (H.D.); 6101116073@email.ncu.edu.cn (Z.X.); 2 College of Qianhu, Nanchang University, Nanchang 330031, China; 3 National Research Council Canada, Ottawa, ON K1S 5B6, Canada; Chunsheng.Yang@nrc-cnrc.gc.ca

**Keywords:** machine learning, effective cuckoo search, BP neural network, IEC-three ratio method, power transformer PHM, fault diagnosis

## Abstract

An emerging prognostic and health management (PHM) technology has recently attracted a great deal of attention from academies, industries, and governments. The need for higher equipment availability and lower maintenance cost is driving the development and integration of prognostic and health management systems. PHM models depend on the smart sensors and data generated from sensors. This paper proposed a machine learning-based methods for developing PHM models from sensor data to perform fault diagnostic for transformer systems in a smart grid. In particular, we apply the Cuckoo Search (CS) algorithm to optimize the Back-propagation (BP) neural network in order to build high performance fault diagnostics models. The models were developed using sensor data called dissolved gas data in oil of the power transformer. We validated the models using real sensor data collected from power transformers in China. The results demonstrate that the developed meta heuristic algorithm for optimizing the parameters of the neural network is effective and useful; and machine learning-based models significantly improved the performance and accuracy of fault diagnosis/detection for power transformer PHM.

## 1. Introduction

Prognostic and System Health Management (PHM) generally provides capabilities such as fault detection, fault prediction, and component life tracking to assess product reliability. PHM technologies include sensing, anomaly detection, diagnosis, prediction and decision support for intelligent machinery maintenance and health operation. Taking advantage of advances in sensor technologies, PHM enables a pro-active fault prevention strategy through continuously monitoring the health of complex systems. A power transformer is a piece of equipment that is of great importance to the electronic system. Thus, its performance can have a great impact on the power grid [1,2,3]. Power transformer aging is an important factor leading to grid failure, which can also cause three main fault types in transformers: electrical, mechanical, and thermal failure. Among them, mechanical failure ranks first [4,5]. Therefore, it is critical to improve the accuracy of fault diagnosis of power transformers [6,7].

Some traditional methods for fault diagnosis of transformers such as dissolved gas analysis (DGA) [8,9,10], short circuit reactance (SCR) [11], and frequency response analysis (FRA) [12] have been widely used in industries. Nevertheless, these methods were limited by the low accuracy of fault diagnosis when the component of the dissolved gas in oil is complicated. High-dimensional fault data on power transformers can lead to the nonlinearity of the whole system, and FRA and SCR in this condition cannot find the real locations of fault, and also cannot provide the information about the types of transformers [11].

The methods of power transformer faults’ diagnosis [13] include mainly the International Electrotechnical Commission (IEC) four-ratio and the three-ratio method, characteristic gas method and so on. However, these methods generate large errors in the diagnosis of power transformers. The accuracy will be greatly reduced when the sample data are too small or there are some outliers the samples. Therefore, artificial intelligence technology with excellent performance is desired to be used in transformer fault diagnosis. Intelligent algorithms based on the DGA data are the widely-used methods in transformer fault diagnosis, especially the back-propagation (BP) neural network [14,15]. The BP neural network can be utilized to find the connection weights and bias to implement accurate diagnostic methods or models for DGA. The updated parameters of BP neural network follow the rule of gradient descending to avoid mistaking the parameters as the optimal parameters.

Nowadays, many smart optimization algorithms and machine learning algorithms have been applied to different domains such as power transformers since these methods have great fault diagnosis performance. There are plenty of power transformer fault diagnoses and other cutting-edge research. In the fault diagnosis of power transformers, various intelligent and machine learning methods are used to detect the state of transformers.

As for power transformer fault diagnosis, Khmais et al. [16] developed a fault classification method of power transformer based on support vector machine (SVM) using train data to build a multi-layer SVM classifier. This classifier has superior performance in identifying transformer fault types. Li et al. [17] presented an intelligent method for power transformer fault diagnosis based on selected gas ratio and SVM. They used a genetic algorithm (GA) to obtain the optimal dissolved gas ratio (ODGR) for DGA ratio selection and support vector machine parameter optimization. Three and four-digit coding with faulty information and fuzzy logic is used to improve the result by Hooshmand et al. [18]. The method has been applied to the diagnosis of dissolved oil in the transformer. Wang [19] developed a new transformer fault diagnosis method based on a probabilistic neural network (PNN) and dissolved gas analysis. A hybrid evolutionary algorithm based on particle swarm optimization (PSO) and BP is used to optimize the parameters of PNN. In order to solve the problem of power transformer accidents, Trappey et al. [20] developed an intelligent engineering asset management system. Data-driven models are used to detect potential faults in transformers. The Principal component analysis (PCA) and BP-Artificial Neural Network (BP-ANN) are used as prediction models to carry out this task. Zheng et al. [21] proposed a transformer solubility prediction method based on PSO and least squares support vector machine (LS-SVM). The results demonstrated that the method is superior to BPNN, Generalized Regression Neural Network (GRNN), Radial Basis Function Neural Network (RBFNN) and Support Vector Regression (SVR) methods.

With regard to another piece of equipment detected by novel methods, Zhou et al. [22] presented a method of intelligent fault diagnosis based on ontology and FMECA (Failure Mode, Effects and Critically Analysis) for the fault diagnosis of wind turbines. This method realizes the knowledge sharing between deep knowledge and shallow knowledge, improves the fault diagnosis ability and makes a better decision for the diagnosis system. In order to improve the efficiency and accuracy of transient probability analysis of flexible mechanisms, a dynamic network method (DNNM) based on Improved PSO/Bayesian regularization (BR) is proposed by Song et al. [23]. The results show that the method improves the computational efficiency and provides a meaningful insight for flexible mechanisms. In order to address the problem of rolling bearing tip under complex working conditions, it is often affected by mechanical and electrical system faults. Therefore, Lu et al. [24] proposed a deep learning method based on a convolutional neural network (CNN). Evssukoff and Gentil [25] proposed a recursive neural fuzzy system for fault detection and isolation in nuclear reactors. It generates good performance in detecting and isolating various security related faults.

This paper is extended from the DPDC 2008 conference, entailed, “Cuckoo Search Optimized NN-based Fault Diagnosis Approach for Power Transformer PHM” [26]. Based on the recommendation from the SPSC 2008 committee, we extensively rewrote the paper by extending the experiments and providing more validation results obtained from real transformer sensor data collected in a smart grid. The main contributions are as follows: (1) to develop machine learning-based models for transformer PHM, we proposed a novel method to enhance the cuckoo search algorithm for optimizing the parameters of multi-layer back-propagation neural network for fault diagnosis of a power transformer. (2) We introduce the important factors such as improvement rate (IR) to update the function of cuckoos (solutions). (3) Given the mutation of the process of finding optimal solution, we consider that the mutation of solution *x*, which is controlled by mutation probability $P_{m}$. (4) We evaluated the developed machine learning-based PHM models by using the real operational data collected from power transformers in a smart grid. The results demonstrated the high performance of the PHM models for transformer fault diagnosis.

The paper is organized as follows. After the Introduction section, Section 2 presents the machine learning-based method, using a Cuckoo search algorithm to optimize the BP neural network for power transformer fault diagnosis. Section 3 introduces the developed machine learning-based model for power transformer fault diagnosis; Section 4 presents the experiments and the results; Section 5 discusses the results and draws the conclusions.

## 2. Methods

### 2.1. Modified Cuckoo Search (MCS) Algorithm

Cuckoo Search Algorithm (CS) is a nature-inspired meta heuristic algorithm which imitates parasitic brood behavior of cuckoos [27]. To simulate the behavior of cuckoo nesting, the CS algorithm sets three rules. The cuckoo produces an egg each time, which represents a solution to the problem, and randomly places the eggs in a nest for hatching. In addition, the number of nests is fixed and set a value $P_{a}\in \left(0,1\right)$ to describe the probability that the nest owner finds the that the egg is a foreign egg. CS is enhanced by the Levy flight so that CS can explore global space and local space of solution and combine them with local search and global search mechanisms that make itself efficient [28]. In addition, important parameters $P_{a}$ and step-size α of CS algorithm in fine-tuning of solution vectors are used to adjust the convergence rate of the algorithm. However, the standard CS algorithm uses a constant value for these parameters by the experience. Unquestionable parameter setting and constant parameters during iterations will decrease the performance of CS algorithm [29].

Thus, in order to improve the ability and overcome disadvantages, a modified Cuckoo Search Algorithm (MCS) is proposed in [30], which the main task is to implement the iterative process in which parameters $P_{a}$ and α are updated via function in the appropriate range.

In order to use feedback information during evolution, parameters $P_{a}$ and α are set as proportional to the improvement rate (*IR*). In addition, the *IR* can be computed by

(1) $$IR=\frac{\sum_{i=1}^{NN}NI_{i}}{NN},$$

where

(2) $$NI_{i}=\left\{\begin{array}{c} 1,\;f\left(x_{i}^{t+1}\right)<f\left(x_{i}^{t}\right),\\ 0,\;otherwise, \end{array}\right.$$

where *NI* is the number of improvement of solutions. *f* is the fitness function we set. *NN* is the total population size.

The discovery probability $P_{a}$ and step size α are dynamically updated as follows:

(3) $$P_{a}=P_{amin}+\left(P_{amax}-P_{amin}\right)\cdot IR^{m},$$

(4) $$\alpha =\alpha_{min}+\left(\alpha_{max}-\alpha_{min}\right)\cdot IR^{n},$$

where $\alpha_{max}$ and $\alpha_{min}$ are the maximum and minimum values of step size α, respectively. $P_{amax}$ and $P_{amax}$ are the maximum and minimum values of discovery probability $P_{a}$, respectively. *m* and *n* are nonlinear factors for adjusting the speed of change of the control parameters.

There are two different strategies in MCS for exploration and exploitation. The first strategy uses Mantegna’s algorithm [31] as follows:

(5) $$x_{i}^{t+1}=x_{i}^{t}+\alpha L\left(s,\lambda \right),$$

where

(6) $$L\left(s,\lambda \right)=\frac{\lambda \Gamma (\lambda )sin(\pi \lambda /2)}{\pi }\frac{1}{s^{1+\lambda }},\;\left(s\gg s_{0}>0\right).$$

Here, *L* is the characteristic scale of the data set. α in Equation (5) is the step size. λ is the Levy exponent which controls the scale of distribution. *s* is the step size that can be computed as follows:

(7) $$s=\frac{U}{{\left|V\right|}^{1/\lambda }},$$

where $U∼N(0,\sigma^{2})$, V∼N(0,1). In addition, $\sigma^{2}$ can be calculated as follows:

(8) $$\sigma^{2}={\left[\frac{\Gamma (1+\lambda )}{\lambda \Gamma ((1+\lambda )/2)}\cdot \frac{sin(\pi \lambda /2)}{2^{(\lambda -1)/2}}\right]}^{1/\lambda }.$$

The second strategy is to attract the closest individuals of the current solution and conduct a global random walk.

These two strategies are randomly selected by the switching probability $P_{c}$, and the second strategy is described as follows:

(9) $$x_{i}^{t+1}=x_{i}^{t}+t_{1}\cdot \left(x_{q1}^{t}-x_{i}^{t}\right)+T\cdot \left(x_{q2}^{t}-x_{q3}^{t}\right),$$

where the integers q1, q2 and q3 represent three mutually different indices randomly selected in the range [1, 2, …, *NN*], which are different from the integer *i*. *T* is scaling factor and $t_{1}$ is a random number within the interval [0, 1].

In addition, to strengthen the global search capability of MCS, a mutation strategy is also introduced through mutation probability $P_{m}$. In addition, the mutation is as follows:

(10) $$x_{ik}^{t+1}=x_{ik}^{t}+t_{2}\cdot \left(x_{ik}^{t}-x_{jk}^{t}\right),$$

where $t_{2}$ is a random number in the interval [−1, 1]. *i* and *j* are different integers selected within the range [1, 2, …, *NN*]. *k* is an integer within the range [1, 2, …, *D*], *D* is the solution space dimension.

In addition, the parameter Pa is to judge the probability of hosts finding exotic birds’ eggs. It can determine whether to generate the next new nest. At this time, the location update equation is given by

(11) $$x_{i}^{t+1}=x_{i}^{t}+r_{1}\cdot \left(x_{q1}^{t}-x_{q2}^{t}\right)+r_{2}\cdot \left(x_{best}^{t}-x_{q3}^{t}\right),$$

where the integers $q_{1}$, $q_{2}$ and $q_{3}$ denote three different integers. $r_{1}$ and $r_{2}$ are randomly generated numbers in [0, 1].

The initial location of MCS can be expressed as:

(12) $$x_{i}=Lb+rand\times \left(Ub-Lb\right),$$

where the Ub and Lb are the upper and lower bounds of the search space, respectively.

The pseudo-code of modified CS algorithm is shown as Algorithm 1.

**Algorithm 1:** Pseudo-code of the modified CS algorithm

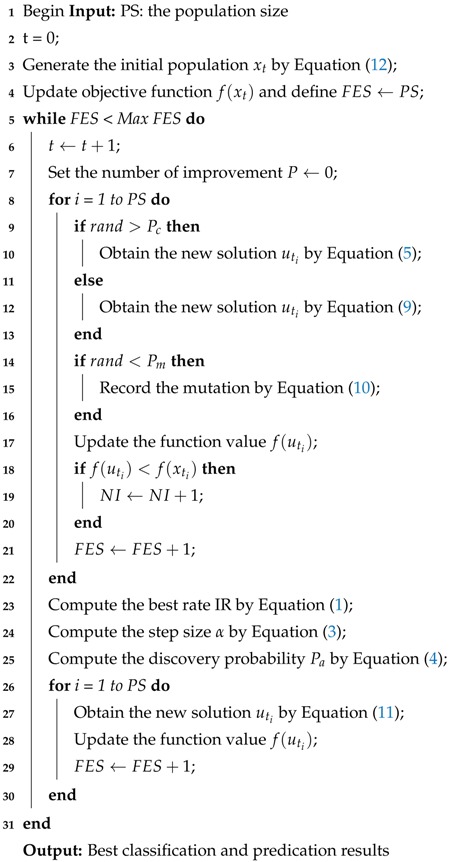



### 2.2. Back-Propagation (BP) Neural Network

Back-propagation (BP) neural network is a multi-layer feed-forward neural network, which belongs to an uncertain nonlinear mathematical model [32,33,34]. The BP network consists of an input layer, hidden layer and output layer. The two processes of forward propagation and back propagation are of great importance to the BP neural network [35,36]. The BP network can have better performers in classification and prediction because of the combination of these two processes. In the forward propagation, the data are passed through the input layer and combined with the hidden layer weights and thresholds to calculate layer by layer, and finally reach the output layer to obtain the classification result. In back propagation, when the output in the output layer does not comply with expectations, the error signal will propagate back. It uses an error gradient descent algorithm to reduce the mean square error (MSE) between the network output value and the actual output value, and the network adjusts the weights and thresholds layer by layer from the output layer to all hidden layers. Finally, the corrected result is output to the output layer.

**Figure 1:**
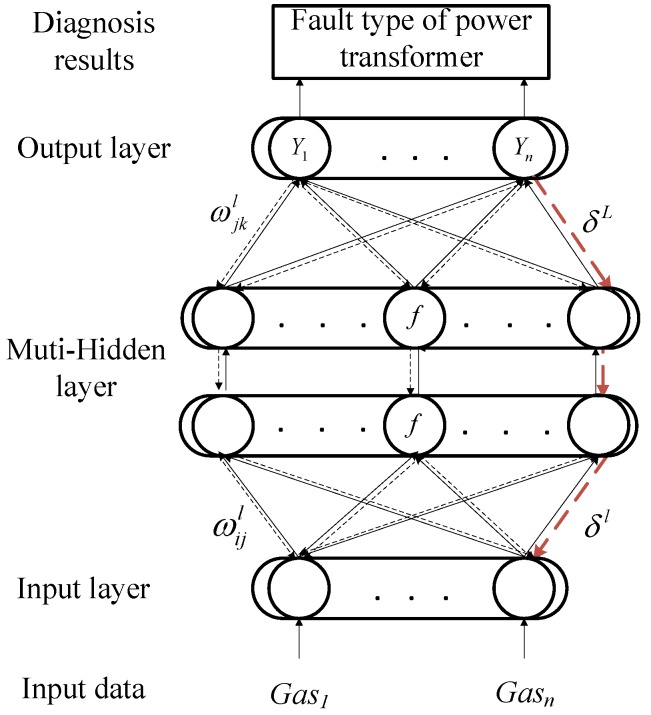
BP neural network model structure.

- Feed-forward
  After recording the input value vector *x*, the activation $a^{l}$ in the input layer *l* can be computed in a simple and compact vectorized form:
  (13) $$a^{l}=f\left(w^{l}a^{l-1}+b^{l}\right)\;\left(l=2,3,\ldots ,L\right),$$
  where $w^{l}$ and $b_{j}^{l}$ are the weight and the bias between the ${\left(l-1\right)}^{}$th and the $l^{}$th layer.
  To set the corresponding activation, this paper uses the most popular sigmoid function:
  (14) $$\sigma \left(x\right)=\frac{1}{1+e^{-x}}.$$
  The quadratic error criterion function of sample *n* is *C*:
  (15) $$C=\frac{1}{2n}\sum_{x}{∥y\left(x\right)-a^{L}\left(x\right)∥}^{2}.$$
- Back-Propagation
  While reaching the layer *L*, the output error $\delta^{L}$ can be calculated by
  (16) $$\delta^{L}=\nabla_{a}C⨀\sigma^{\prime }\left(z^{L}\right).$$
  $\nabla_{a}C$ contains the rate of C changing. ⨀ denotes the entry-wise product of two vectors. Subsequently, the error in the next layer is
  (17) $$\delta^{l}=\left({\left(w^{l+1}\right)}^{T}\delta^{l+1}\right)⨀\sigma^{\prime }\left(z^{l}\right),$$
  where ${\left(w^{l+1}\right)}^{T}$ is the transpose for the ${\left(l+1\right)}^{}$th layer. $⨀\sigma^{\prime }\left(z^{l}\right)$ is the Hadamard product within the interval.
  According to the error gradient descent method, the threshold can be calculated as follows:
  (18) $$\frac{\partial C}{\partial b_{j}^{l}}=\delta_{j}^{l}.$$
  Any weight in the network is:
  (19) $$\frac{\partial C}{\partial w_{jk}^{l}}=a_{k}^{l-1}\delta_{j}^{l}.$$
  By combining Label (11) with Label (12), the error goes backward through the activation function in layer *l*.
  The BP neural network model structure can be seen in Figure 1.

### 2.3. MCS Optimized BP Neural Network (MCS-BP)

The fault diagnosis of a power transformer based on an MCS optimized BP neural network can be used as a comprehensive diagnosis platform, which combines the data of gas in oil with the detection system, and then obtains good results by supervised learning methods.

**Figure 2:**
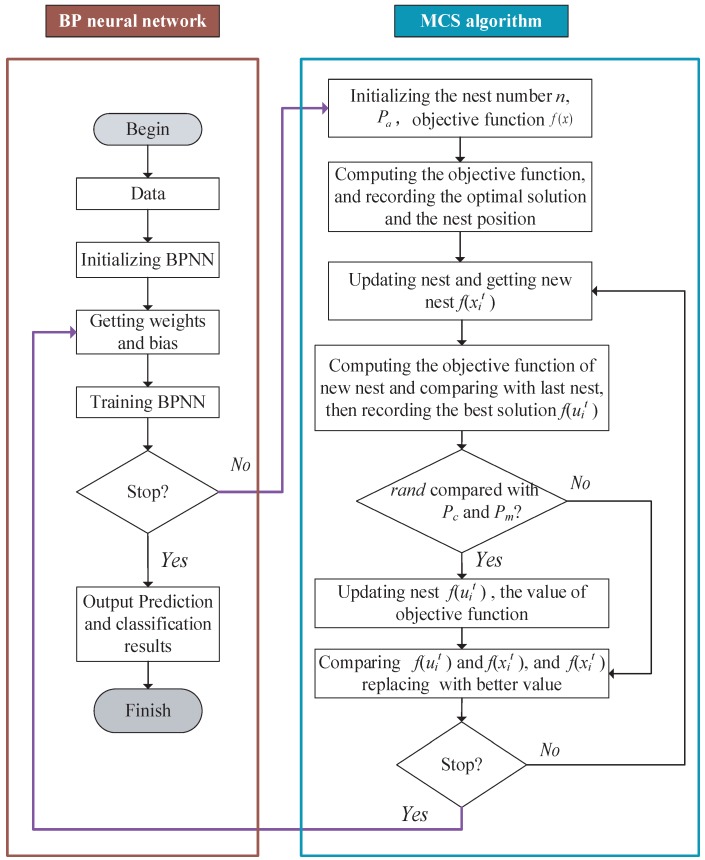
The flowchart of MCS-BP.

As shown in Figure 2, MCS optimizes the block diagram of BP neural network. The following are the main steps:

Step 1: At first, use an IEC three-ratio method to process the features of DGA data of power transformer.

Step 2: Randomly choose the different types of faults of power transformers into the neural network.

Step 3: Initialize the parameters of the BP neural network.

Step 4: Initialize the modified cuckoo search size $x_{i}\;\left(i=1,2,\ldots ,N\right)$, population size *N*, switching probability $P_{c}$, mutation probability $P_{m}$, and value of step size $\alpha_{0}$, maximum value of step size $\alpha_{max}$, minimum value of step size $\alpha_{min}$, maximum value of discovery probability $P_{amax}$, minimum value of discovery probability $P_{amin}$, nonlinear factor *m* and *n*, scaling factor F and the fitness function f(x). The fitness function we used in this paper is the mean square error (MSE), as follows:

(20) $$f\left(x\right)=\frac{1}{2n}\sum_{i=1}^{N}{\left(Y_{i}-O_{i}\right)}^{2},$$

where $Y_{i}$ is the measure value and $O_{i}$ is the predicted result.

Step 5: Calculate the fitness value of the initial nest via the fitness function, and then select the current optimal solution in the solution space.

Step 6: Generate a random number K1 and compare with $P_{c}$. Compare K1 and $P_{c}$, if $K1>P_{c}$, update nests $x_{i}^{t+1}$ via Equation (5), otherwise by Equation (9).

Step 7: Generate a random number K2 and compare with $P_{m}$ of MCS. If $K2<P_{m}$, perform the mutation via Equation (10); otherwise, it is unchanged.

Step 8: Calculate the updated solution’s fitness value and update the discovery probability $P_{a}$ and the step size α via Equation (3) and Equation (4).

Step 9: Generate a random number K3 and compare with $P_{a}$. If $K3>P_{a}$, update nests $x_{i}^{t+1}$ via Equation (11), or do not change. Compare the last fitness values with new birds’ nests, keep the optimal bird’s nest as the contemporary best nest $x_{b}$.

Step 10: If it can reach the maximum iteration condition, proceed to the next step, or return to Step 6.

Step 11: Substitute the optimized weights and bias of the BP neural network.

Step 12: Input the test set into the trained BP neural network to get the classification output.

## 3. MCS-BP for Power Transformer Fault Diagnosis Platform

**Figure 3:**
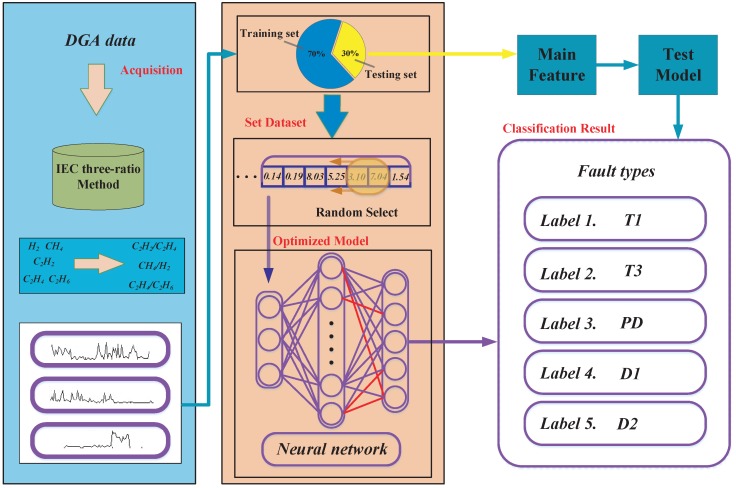
Structure flow of the power transformer fault diagnosis process.

In this paper, power transformer fault diagnosis is mainly divided into four parts: data collection and preprocessing, segmentation of data set, neural network model-training, and comparison between test set output and train set output, as is shown as Figure 3.

In Figure 3, firstly power transformer DGA data will be processed in feature selection via the IEC three-ratio method. This procession can be seen in Table 1. Then, 70% of the data can be used in the training model, which has been sorted randomly to ensure the train set and test set containing all types of faults. The other 30% of the data is utilized to test the optimized model. In this study, we test five types of faults of power transformers, which are the thermal faults *T* > 700 °C, thermal faults *T* < 300 °C, high energy discharge, low energy discharge and partial discharge. It can be seen as Table 2, and each group of data is balanced. There are 109 sets of data.

Through this optimization model, the potential faults of power transformers can be predicted and classified.

## 4. Experimental Setup and Results

### 4.1. Experimental Setup

In order to evaluate the performance of the proposed method in power transformer fault diagnosis, we obtain real-world data to implement experiments. By IEC three-ratio processing 109 sets of DGA data, the feature-filtered data sets are obtained, some of which are shown in Table 3. In this paper, the neural network is used as the basic classifier, so the encoding method of data is shown in Table 4.

The MCS-BP method is compared with other excellent predictive classifiers BP, CS-BP, Multi-Verse Optimizer-Multi-Layer Perceptron (MVO-MLP), PSO-BP, GA-BP, PNN and SVM, respectively. Firstly, the accuracy and error rate of the algorithm of MCS-BP is compared with CS-BP and BP, and the superiority of the algorithm of MCS in the optimization of a neural network is proved. Secondly, by comparing the MCS-BP algorithm with other machine learning algorithms and optimization algorithms, it is proved that the algorithm has strong robustness and classification performance.

### 4.2. Experimental Results

Firstly, the MCS-BP algorithm and CS-BP algorithms are compared. In this experiment, the parameters of the BP neural network are the same, its structure is 3-4-5, three-dimensional input, five-dimensional output, for MCS and CS, the MCS algorithm sets the Levy exponent λ to 1.5, the step size $\alpha_{0}$ to 0.1, $\alpha_{max}$ to 0.5, $\alpha_{min}$ to 0.05, the discovery probability $P_{amax}$ to 0.5, $P_{amin}$ to 0.1, $P_{c}$ to 0.3, $P_{m}$ to 0.3, *m* to 0.5, and *n* to 0.5. The CS algorithm sets the discovery probability $P_{a}$ to 0.25, λ to 1, and the step size α to 0.4. The accuracy of classification is shown in Table 5. For five types of fault outputs, MCS-BP has a high recognition rate of 97.14% with asterisk, and the classification recognition rate of different fault types is higher than or equal to the BP and CS-BP algorithms.

As shown in Table 6, the training set and test set MSE of MCS-BP are the smallest. Compared with the other two algorithms, the performance of MCS is better than that of normal BP and standard CS.

Next, we use many different algorithms to further evaluate the classification performance of MCS-BP. The GA sets the crossover probability to 0.7 and the mutation probability to be 0.01. The PSO algorithm sets the maximum speed to 1, the minimum speed to −1, the solution space is [−5, 5] and the learning factor to 1.49445. The recognition rate is shown in Table 7. From Table 7, it can be found that the recognition rate and Micro F1-score of MCS-BP is obviously higher than that of other algorithms, and for fault type T1 and PD, the classification recognition rate of other algorithms is very low, and the diagnosis rate of MCS-BP is still very high. The error rate comparison is shown in Table 6, from which we can see that the MSE of MCS-BP is still the smallest, indicating the excellent performance of MCS-BP. From Table 8, we further know that the performance of the classification of MCS-BP is better than other models via each fault type F1-score and the Macro F1-score (98.46%).

**Figure 4:**
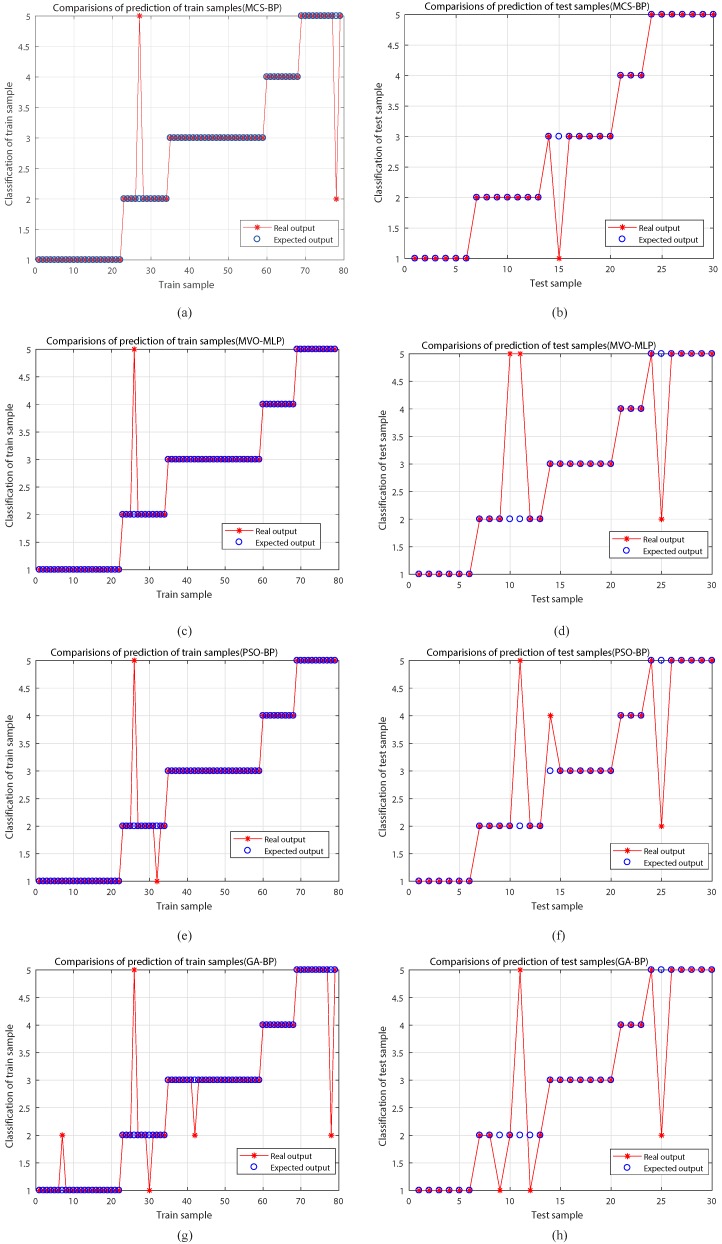
The classification results of different models. (**a**), (**c**), (**e**) and (**g**) represent the results of train sample classification for different methods, respectively. (**b**), (**d**), (**f**) and (**h**) are the results of test sample classification for different methods, respectively.

In order to further evaluate the performance of MCS-BP, we plot the output results of different algorithms, such as Figure 4, Figure 4a,c,e,g for the output of training set and the layout of predicted classification results, and Figure 4b,d,f,h for the output of test set classification. Its test set classification effect is not ideal; however, the MCS-BP (a), (b) shows a good recognition rate, and (b) shows that only the third fault type judgment is an error.

By Figure 4, we know that the developed method is better than other algorithms with respect to stability as the test data results can reflect that our model won’t fall into the problem of over-fitting, and both the train sample and test samples have great classification results. It indicates that this model is helpful for fault diagnosis of power transformer because it can give a suitable decision for which the fault type of power transformer is contained.

**Figure 5:**
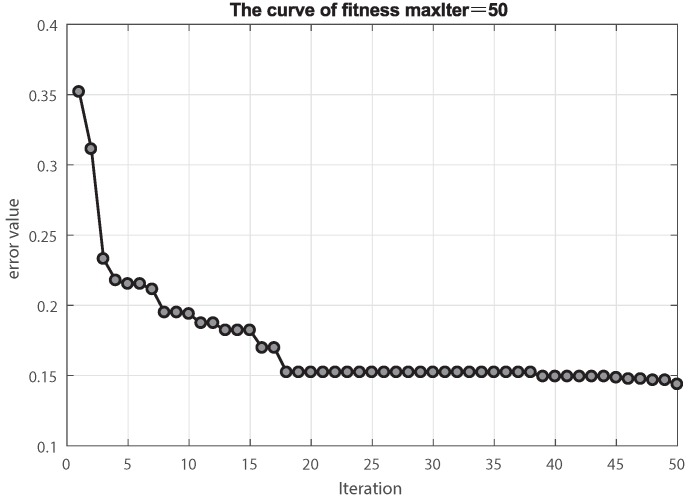
The curve of fitness of MCS-BP.

**Figure 6:**
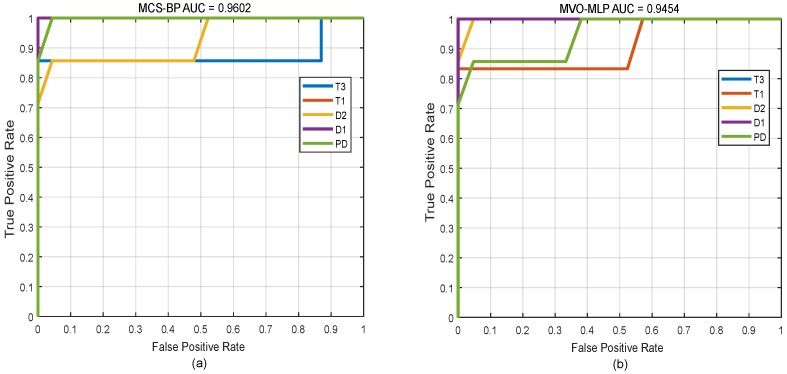

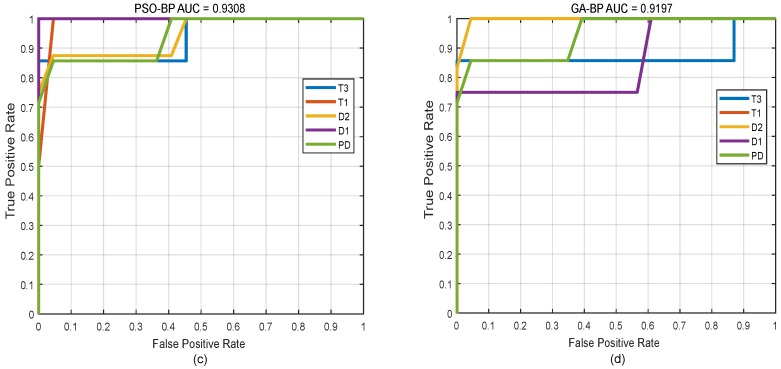
The curve of ROC-AUC of different models. (**a**–**d**) represent the receiver operating characteristic curve with AUC for MCS-BP, MVO-MLP, PSO-BP and GA-BP, respectively.

In addition, to clarify the optimization effect of MCS on BP during the iteration process, we consider the variation of the fitness (error) of the MCS, as shown in Figure 5. From generation 1 to generation 50, the fitness value of MCS in the process of optimizing neural network decreases rapidly in generations 1 to 4, which shows that the efficiency of global optimization and local optimization of MCS is very high. Then, it falls into a local extremum in generation 18 or so. However, after a period of iteration, it jumps out of a local extremum point and continues to search for optimization. In addition, we completed the Receiver Operating Characteristic (ROC) Curve, which is shown in Figure 6, and obtain the value area under the curve (AUC). Both of them can prove that our model has great performance of classification for power transformers. For Figure 6a, we can see that each fault class can be classified well.

## 5. Conclusions

In this paper, we propose a machine learning-based method, CS optimized BP neural network model for power transformer fault diagnosis. This algorithm can adjust the search step of solution space adaptively to find a better global optimal solution, and the fitness value of each solution is utilized to build the mutation probability to avoid local convergence. In addition, the MCS enhances the exploitation capacity and convergence rate. We conducted the experiments to validate the developed models by using 109 sets of real-world data collected from power transformers. Compared with other algorithms, experimental results show that the MCS method we developed outperformed other methods and can converge to the optimal solution for most test cases.

To validate the machine learning-based models or methods for fault diagnosis, more extensive experiments and more advanced metrics and evaluation tools are in high demand. This will be our future work. We will continue to enhance the performance of the algorithms and models and evaluate the performance of the models under different circumstances of the error rate and operating efficiency, using other evaluation tools and metrics.

## Figures and Tables

**Table 1:** Diagnosis using the three-ratio method (IEC 60599) [37].

Fault Type	$C_{2}H_{2}/C_{2}H_{4}$	${\mathrm{CH}}_{4}/H_{2}$	$C_{2}H_{4}/C_{2}H_{6}$
PD	<0.1	<0.1	<0.2
D1	>1	0.1–0.5	>1
D2	0.6–2.5	0.1–1	>2
T1	NS	>1/NS	<1
T2	<0.1	>1	1–4
T3	<0.2	>1	>4

**Table 2:** Fault type used in analysis.

NO.	Fault Type	Fault Type Code
Fault 1	Thermal faults *T* > 700 °C	T3
Fault 2	Thermal faults *T* < 300 °C	T1
Fault 3	High energy discharge	D2
Fault 4	Low energy discharge	D1
Fault 5	Partial discharge	PD

**Table 3:** Statistical data of partial samples.

$C_{2}H_{4}$	$H_{2}$	$C_{2}H_{6}$	Fault Type
0.019	0.0899	2.157	T1
0.029	0.231	2.654	T1
0.0246	0.9655	8.2797	T3
0.0541	1.2551	8.9697	T3
1.38	0.211	5.396	D2
0.12	0.438	5.664	D2
8.097	2.694	1.752	D1
8.382	2.708	1.768	D1
0	0.041	0.149	PD
0.088	0.052	0.099	PD

**Table 4:** Output target coding of different faults.

	T3	T1	D2	D1	PD
**Coding** **format**	1	0	0	0	0
	0	1	0	0	0
	0	0	1	0	0
	0	0	0	1	0
	0	0	0	0	1

**Table 5:** The comparison of basic methods (* means the best result in the table).

Fault Type	Accuracy Rate (%)		
	BP	CS-BP	MCS-BP
T3	100.00	100.00	100.00
T1	100.00	85.71	100.00
D2	85.71	85.71	85.71
D1	100.00	100.00	100.00
PD	0.00	100.00	100.00
Total	77.14	94.29	97.14 *

**Table 6:** Comparison of sample errors.

Model	MSE of Train Sample	MSE of Test Sample
**BP**	0.0330	0.1571
**CS-BP**	0.0053	0.0220
**MCS-BP**	0.0058	0.0204

**Table 7:** The comparison of different methods (* means the best result in the table).

Fault Type	Accuracy Rate (%)					
	MCS-BP	MVO-MLP	PSO-BP	GA-BP	PNN	SVM
T3	100.00	100.00	100.00	100.00	83.33	83.33
T1	100.00	71.43	85.71	57.14	85.71	28.57
D2	85.71	100.00	85.71	100.00	100.00	71.43
D1	100.00	100.00	100.00	100.00	66.67	100.00
PD	100.00	85.71	85.71	85.71	85.71	85.71
Total	97.14 *	91.43	91.43	88.57	78.57	73.81

**Table 8:** The comparison of different methods with F1-score (* means the best result in the table).

Fault Type	Macro F1-Score (%)					
	MCS-BP	MVO-MLP	PSO-BP	GA-BP	PNN	SVM
T3	100.00	100.00	100.00	100.00	100.00	90.91
T1	100.00	92.31	60.00	92.30	44.44	44.44
D2	92.30	100.00	100.00	92.30	100.00	83.33
D1	100.00	100.00	100.00	100.00	100.00	100.00
PD	100.00	92.30	92.30	92.30	92.30	92.31
Macro F1-score	98.46 *	96.92	90.46	95.38	87.35	82.20

## Abbreviations

PHM	Prognostic and health management
MCS	Modified Cuckoo Search Algorithm
BP	Back-propagation
MCS-BP	Modified Cuckoo Search Algorithm optimized Back-propagation neural network
DGA	Dissolved gas analysis
GA	Genetic algorithm
PCA	Principal component analysis
PSO	Particle Swarm Optimization
IR	improvement rate
α	step-size
$P_{a}$	discovery probability
*f*	fitness function
λ	Lévy exponent
$P_{c}$	switching probability
$P_{m}$	mutation probability
$a^{l}$	activation functions
C	cost function
$\sigma^{L}$	output error
MVO	multi-verse optimizer
MLP	multi-layer perceptron
PNN	probability neural network
MSE	mean square error
SVM	support vector machine
$x_{i}^{t}$	the nest in *t* generation
$x_{i}^{t+1}$	the nest in *t* + 1 generation
